# Lifestyle patterns and their associations with overweight and obesity among children aged 4–9 years in the United Arab Emirates

**DOI:** 10.1017/S0007114525105242

**Published:** 2025-11-14

**Authors:** Farah Naja, Nada Abbas, Katia AbuShihab, Fatima Al Zahraa Chokor, Leila Cheikh Ismail, Ayesha S. Al Dhaheri, Lynda O’Neill, Habiba Ali, Maysm N. Mohamad, Nahla Hwalla, Lara Nasreddine

**Affiliations:** 1 Department of Clinical Nutrition and Dietetics, College of Health Sciences, Research Institute of Medical and Health Sciences, University of Sharjah, Sharjah 27272, United Arab Emirates; 2 Department of Nutrition and Food Sciences, Faculty of Agricultural and Food Sciences, American University of Beirut, Beirut 1107 2020, Lebanon; 3 Department of Public Health, College of Health Sciences, QU Health, Qatar University, Doha 2713, Qatar; 4 Department of Nutrition and Health, College of Medicine and Health Sciences, United Arab Emirates University, Al Ain 15551, UAE; 5 Nestlé Institute of Health Sciences, Nestlé Research, Société des Produits Nestlé S.A., Lausanne 1015, Switzerland

**Keywords:** Lifestyle patterns, Overweight, Obesity, Children, United Arab Emirates

## Abstract

An integrative approach addressing diet and other lifestyle factors is warranted in studying obesity and its related diseases. The objective of this study is to examine the associations of lifestyle patterns with overweight/obesity among children in the United Arab Emirates. Data were derived from a cross-sectional survey of children aged 4–9 years living in Dubai, Sharjah and Abu Dhabi (*n* 426). Dietary intake was collected using a 24-h dietary recall and evaluated with the Healthy Eating Index. The Youth Physical Activity Questionnaire assessed physical activity, while other lifestyle factors included the presence of a live-in household helper, number of electronic devices in the child’s bedroom, eating while watching TV, family dinner frequency, fast-food and breakfast consumption and hours of sleep. Factor analysis was used to identify the lifestyle patterns. Two lifestyle patterns emerged: an unhealthy pattern marked by higher fast-food intake, eating while watching TV, having a live-in household helper and lower family dinners and a healthy pattern with higher physical activity, better Healthy Eating Index, more sleep, micronutrient supplements and breakfast consumption. The healthy lifestyle pattern was linked to a 30 % reduction in overweight/obesity odds (OR = 0·7, 95 % CI: 0·53, 0·93). A healthy lifestyle pattern, characterised by higher physical activity, better dietary quality, adequate sleep and breakfast consumption, is associated with lower odds of overweight/obesity among children in the United Arab Emirates. These findings highlight the importance of promoting comprehensive lifestyle interventions to effectively address childhood obesity in this population.

Paediatric obesity continues to be a critical public health concern worldwide, with its prevalence increasing steadily across many regions^(1,2)^. Data indicate that the Eastern Mediterranean Region harbours a high burden of childhood overweight and obesity^(3)^, particularly within Gulf Cooperation Council countries^(3)^. The early school age has been identified as a critical stage for obesity development^(4)^, often coinciding with notable lifestyle changes among children. For primary school-aged children, family routines often undergo significant adjustments, and children face heightened demands, such as the need for punctuality and for sitting much of the day^(5)^. Their dietary habits also tend to change, with an increasing reliance on snacks and foods available within the school setting^(4)^. In addition, their interest in and use of electronic devices witness sharp increases during this period^(6)^.

It is well-acknowledged that paediatric obesity leads to a wide range of short-term and long-term complications that can impact both physical health and mental well-being. Short-term pathologies of obesity in children might include hypertension, dyslipidaemia, type 2 diabetes mellitus, sleep disorders, mental health disorders and others^(7,8)^. Available evidence from systematic reviews and meta-analyses showed that children with obesity were 26·1 times more likely to develop non-alcoholic fatty liver disease, 1·7 times more likely to report having asthma and 1·85 times more likely to develop depression as compared with those who are of normal weight^(9,10)^. Paediatric obesity can also lead to several long-term consequences that may include the development of various non-communicable diseases including type 2 diabetes^(11–13)^, CVD^(14,15)^, cancer^(16)^and depression^(17)^ during adulthood.

The aetiology of obesity is complex and multifactorial^(18,19)^, being driven by a vast array of intertwined factors that include diet, physical activity, sleep and genetics^(20,21)^. Diet has always been considered a key modifiable factor for obesity, with available evidence showing that increased intake of fast food, soft drinks and added sugar is associated with a higher risk of overweight and obesity in children^(22–24)^. More recently, a plethora of studies^(25–28)^ showed that obesity is related to other lifestyle factors in addition to poor dietary habits. A recent meta-analysis^(25)^ addressing various behavioural risk factors that may affect paediatric obesity showed that, in addition to dietary habits like consuming sugar-sweetened beverages, fast food and fried foods several times a week, children who experienced inadequate sleep and high screen time and those who engaged in computer gaming for more than 2 h/d were more likely to be overweight or obese compared with their peers. In fact, behaviours such as sleep deprivation and excessive TV watching were postulated as determinants of excessive eating and weight gain, due to their involvement in cognitive reward pathways, inhibitory control and appetite-regulatory processes^(29)^. Moreover, behaviours such as insufficient sleep and screen use before bedtime were suggested to influence the risk of obesity through altered metabolic processes, including disrupted energy homeostasis and glucose metabolism^(30–34)^. As such, the investigation of the determinants of paediatric obesity should include a comprehensive evaluation of health behaviours, ideally encompassing diet and other lifestyle factors such as sleep and lifestyle routines.

In support of this holistic approach is the observation that, in real life, certain lifestyle behaviours tend to occur simultaneously and exert a synergetic impact on weight status, thus forming behavioural clusters. For instance, a meta-analysis^(35)^ focusing on the clustering of health-related behaviours among children, adolescents and young adults has identified three predominant behavioural clusters: healthy, unhealthy and mixed. According to this meta-analysis, a healthy cluster is broadly characterised by good diet quality, high physical activity and low sedentary behaviour; an unhealthy cluster is characterised by poor diet quality, low physical activity and high sedentary behaviour, while a mixed cluster includes both healthy and unhealthy behaviours. The meta-analysis results showed that unhealthy and mixed behavioural clusters were associated with higher adiposity rates in comparison with the healthy cluster. This holistic approach can account for the potential bidirectionality and synergy between the different risk factors and provide a more accurate reflection of real-life behavioural patterns, as they may occur according to certain societal norms or within specific regions^(36,37)^.

The United Arab Emirates (UAE) is a developed nation with an estimated gross domestic product of 504 billion US dollars as of 2023^(38)^. Such a high gross domestic product is driven by the rich reserves of oil and gas, as well as the strategic economic diversification^(39)^. The UAE’s swift economic growth and urban development have led to a highly modern and technologically advanced lifestyle^(40)^. However, like most countries of the Gulf Cooperation Council, this was also accompanied by an alarming rise in the burden of obesity and associated non-communicable disease^(41,42)^. A recent review article indicated that the prevalence of obesity was estimated at 17 % among school-age children (5–17 years old) in the UAE^(43)^. The development and implementation of effective culture-specific interventions aimed at curbing the obesity epidemic require a foundational understanding of lifestyle behaviours that increase the risk of paediatric overweight and obesity. It is in this context that this study was undertaken with the aims of (1) identifying and characterising lifestyle patterns among young school-aged children aged 4–9 years in the UAE and (2) examining the associations of these patterns with overweight and obesity in the study sample. The study focuses specifically on children aged 4–9 to capture a developmental stage where diet and lifestyle are largely shaped by the home and family environment, unlike older children whose increasing autonomy and peer influence may confound early-life exposure assessments^(44)^.

## Methods

### Study design

This study utilised data from a previously conducted cross-sectional survey as part of the ‘Feeding Infants and Toddlers Study – Kids Nutrition and Health Study’ that aimed at evaluating food consumption patterns and dietary intakes among children aged 4–12·9 years in the three largest Emirates of the UAE: Abu Dhabi, Dubai and Sharjah. The sampling method employed a stratified random cluster design, where the Emirates represented the strata, and schools, including preschools, were randomly selected as clusters within each stratum. The study received ethical approval from several review bodies, including the Institutional Review Board of the American University of Beirut (ID: SBS-2018-0185, 14 December 2018), the Institutional Review Board of the United Arab Emirates University (ID: FAZ/fa/18-11, 24 January 2019), Dubai Health Authority (ID: DSREC-02/2019_14, 13 May 2019), the UAE Ministry of Health and Prevention (ID: MOHAP/UAQ.REC/010/2018, 25 December 2018), the Ministry of Education in the UAE (MOE) (4 February 2019) and the University of Sharjah (ID: REC-18-10-09-02, 25 November 2018).

### Study population

Children were eligible to participate if they were between 4 and 12·9 years of age and had no medical conditions that could affect their dietary intake or anthropometric measurements, such as metabolic disorders, chronic illnesses or physical disabilities. Children of families who had lived in the UAE for less than 3 years were excluded since short-term residents may retain dietary behaviours shaped by their country of origin and may not reflect sustained exposure to the local food environment, education and health systems, as well as cultural practices in the UAE^(45,46)^. Children whose mothers were under 18 years old were also excluded, since individuals under this age are considered minors. In total, 646 children participated in the original survey, comprising 431 national and 215 non-national children. Further details regarding the study population and the specific sampling techniques are provided elsewhere^(47)^. For the purpose of this study, data of children aged 4–9 years were analysed (*n* 426).

Children were recruited from preschools and schools between June 2019 and March 2020. Following the review and approval of the MOE and the administration of the selected schools, invitation letters were sent to the parents of students within the targeted age group at the school. One-to-one interviews were scheduled with interested parents, during which the main caregiver was asked to review and sign the informed consent form. In this study, all the main caregivers were the mothers of the children.

### Data collection

Through one-to-one interviews, trained nutritionists obtained anthropometric measurements for both the mother and the child using standardised protocols and equipment. Height was measured without shoes, using a stadiometer (Seca 217), and measurements of weight were obtained in light clothing using a clinical balance (Seca 874). The BMI of mothers was calculated as the ratio of weight (kg)/height (m)^2^. Children’s weight status was classified using the age and sex-specific cutoffs of the BMI-for-age Z-score (BAZ), as per the WHO Child Growth Criteria^(48)^, as follows: children aged 4–5 years, wasting <−2; normal status −2 ≤ BAZ ≤ +1; possible risk of overweight +1 ≤ BAZ ≤ +2; overweight +2 ≤ BAZ ≤ +3; and obese as BAZ > +3; children aged 5–9 years, wasting < −2; normal status −2 ≤ BAZ ≤ +1; overweight status +1 ≤ BAZ ≤ +2; and obese status of BAZ > +2^(48)^.

A multicomponent questionnaire was used by the nutritionists to obtain information regarding the child’s lifestyle behaviours. These behaviors included dietary intake and physical activity, in addition to other lifestyle characteristics such as the daily numbers of hours slept, whether or not the child usually eats while watching TV, the presence/absence of a live-in household helper, the number of electronic devices in the child’s bedroom, whether or not the child is taking micronutrients supplements or not, in addition to the frequency of dinner consumption with the family and the frequency of fast-food consumption. The frequency of family dinners was assessed with the question: ‘How many nights a week does (CHILD) typically sit down to have dinner together with the family?’ Responses were categorised as never or rarely (0–2 nights/week), sometimes (3–6 nights/week) and always (every night). Fast-food consumption was assessed with the question: ‘About how often does (CHILD) eat fast food?’ Response options ranged from every day to never and were categorised for analysis based on frequency.

The five-step multiple-pass 24-h dietary recall (24-HR) was used to assess the child’s dietary intake^(49)^, with mothers as proxies. The multiple pass 24 HR collects a detailed description of all foods consumed by the child during the previous day, including the type of food, its quantity and cooking method or brand. In addition, information about the meals (breakfast, lunch, dinner) and snack consumption was obtained. For the estimation of nutrient intake, the Nutritionist Pro^TM^ Software was used. For mixed and traditional dishes, local recipes were added to the Nutritionist Pro software as single food items. Within the software, the US Department of Agriculture database was selected; additional food composition data were retrieved from the food composition tables for the Middle East^(50)^, as well as from nutritional information on the product packaging and websites when applicable, and data from published studies that reported on specific traditional UAE dishes^(51,52)^. Using data from the 24-HR, information regarding whether the child has consumed breakfast or not was obtained.

To evaluate the quality of the diet, the Healthy Eating Index (HEI) as described by Krebs-Smith *et al.* ^(53)^ was calculated. This index consists of thirteen components divided into two categories: adequacy and moderation (Appendix 1). Adequacy components measure whether individuals consume enough of the recommended foods, which include fruits, vegetables, whole grains, dairy, protein and fatty acids, while the moderation components assess the intake of foods that should be limited, such as refined grains, sodium, added sugars and saturated fats. Each component is scored based on its intake level, with higher scores allocated for a higher consumption of the adequacy components and lower consumption of the moderation components. Component scores range from 0 to 5 or 0 to 10 and are summed to give a total HEI between 0 and 100, with higher values indicating a healthier diet, with the following categorisation: poor (< 60), moderate (60–80) and good (> 80)^(53)^.

The Youth Physical Activity Questionnaire was used to examine the children’s physical activity levels^(54)^. This questionnaire assesses the frequency and duration of the child’s various physical activities over the past 7 d. Based on the WHO recommendations for physical activity, children were categorised as either meeting or not meeting the requirements for their age group. These requirements were ‘at least 180 min in a variety of types of physical activities at any intensity, of which at least 60 min is moderate-to-vigorous intensity physical activity, spread throughout the day’ for ages 3–4 years old children and ‘at least an average of 60 min/d of moderate-to-vigorous intensity, mostly aerobic, physical activity, across the week’ for ages 5 to years^(55)^.

### Data analysis

Data were analysed using Stata version 15. Statistical power analysis was carried out using G ^*^ Power 3.1. The availability of 426 participants in the present study was sufficient to achieve a statistical power of 80 % to detect an association between lifestyle patterns and obesity (defined as overweight/obese *v*. normal weight), assuming an OR of 0·6, with a two-tailed test and a significance level (*α*) of 0·05. As such, given the available sample size and the expected strength of association, the study had an 80 % probability of correctly rejecting the null hypothesis (i.e. identifying a true association, if one exists) while maintaining a 5 % risk of committing a Type I error. The sample size calculations for the original survey study are reported in detail elsewhere^(56)^.

Descriptive statistics were used to summarise the baseline characteristics of the study participants. Continuous variables were presented as means with standard deviations (sd) (mean (sd)), while categorical variables were summarised as frequencies and percentages. Factor analysis with varimax rotation was employed to derive lifestyle patterns based on selected lifestyle characteristics, including diet (as assessed by the HEI), fast-food consumption, breakfast consumption, consuming dinner with the family, eating while watching TV, presence of a live-in helper, physical activity, use of supplements, presence of an electronic device in the child’s bedroom and sleep. The suitability of the data for this technique was assessed using the Kaiser–Meyer–Olkin test (0·505) and the *χ* ^2^ Bartlett test of sphericity (*P* < 0·001). While the Kaiser–Meyer–Olkin value meets the minimum threshold for factor analysis, it falls at the lower edge of acceptability, indicating only modest adequacy for identifying latent constructs. Factor loadings were used to interpret and label the identified patterns. While eigenvalues greater than 1·0 (Kaiser’s criterion) were considered, the number of factors retained was determined following a multi-criterion approach, which included visual inspection of the scree plot to identify the point of inflection (Cattell’s criterion), the magnitude of eigenvalues and the interpretability and conceptual meaningfulness of the factor structure^(57)^. By integrating statistical and theoretical considerations, we ensured that the retained factors were both robust and relevant to the study context. Simple and multiple logistic regression models were used to assess the associations between the derived lifestyle patterns and the odds of being overweight or obese, as indicated by the classification of the BAZ, as the dependent variable. Basic characteristics associated with obesity at the bivariate level were included in the multiple logistic regression model. OR with 95 % CI were reported. A *P*-value of < 0·05 was considered statistically significant.

## Results

The study included 426 children with a mean age of 6·18 years (sd = 1·4). Mothers had a mean age of 35·82 years (sd = 6·09) and a mean BMI of 28·12 kg/m^2^ (sd = 5·08) (Table 1). The sex distribution of the children was nearly equal (47·9 % male, 52·1 % female). Mean BAZ was 2·31 (±0·92), with children distributed as follows: wasted (*n* 44, 10·3 %), normal (*n* 333, 78·2 %), overweight (*n* 28, 6·6 %) and obese (*n* 21, 4·9 %). Emirati children made up 64·3 % of the study sample. Among mothers, 51·4 % had up to high school education, and 53·8 % were unemployed. Fathers had similar education levels (48·6 % up to high school), and 93·6 % were employed. Most families (89·3 %) had a crowding index of ≥ 1 person/room. Regarding income, 35 % earned up to 30 000 AED (Arab Emirates Dirhams), 18·8 % above 30 000 AED and 46·2 % either didn’t know or refused to disclose (Table 1).

**Table 1.** Characteristics of study participants (*n* 426) (Mean values and standard deviations; numbers and percentages)

	Mean	sd
Child’s age (years)	6·18	1·4
Mother’s age (years)	35·82	6·09
Mother’s BMI (kg/m^2^)	28·12	5·08
	*n*	%
Child’s sex		
Male	204	47·9
Female	222	52·1
Child’s BAZ^*^		
Mean	2·31	
sd	0·92	
Wasted/normal	377	88·5
Overweight/obese	49	11·5
Nationality		
Emirati	274	64·3
Non-Emirati	152	35·7
Mother’s education		
Lower education/intermediate/high school	219	51·4
University or higher	207	48·6
Mother’s employment status		
Unemployed	229	53·8
Employed	197	46·2
Father’s education		
Lower education/intermediate/high school	205	48·6
University or higher	217	51·4
Father’s employment status		
Unemployed	27	6·4
Employed	395	93·6
Crowding index		
< 1 person/room	45	10·7
>= 1 person/room	377	89·3
Family income		
Up to 30 000 AED	149	35
30 000 AED and above	80	18·8
Don’t know/refuse	197	46·2

AED, United Arab Emirates dirham; BAZ, BMI-for-age Z-score.

* The normal category included the ‘at risk for overweight’ for the age group 4–5 years.

Table 2 summarises the lifestyle characteristics of the study participants. Most households (60·6 %) had a live-in household helper. Electronics were present in 27·5 % of children’s bedrooms. A large percentage (65 %) reported eating while watching TV, and 80·7 % had dinner with family every night. Micronutrient supplements were used by 23·9 % of the study sample. Fast food was consumed once per week by 44·7 %, while 21·2 % reported consuming it more often. Breakfast was consumed, during the previous day, by 97·7 % of the participating children. Only 40·8 % met the physical activity recommendations. Average sleep was estimated at 10·37 h/d, and the mean HEI was 55·15, with 60·3 % scoring in the poor range (0–59). (Table 2)

**Table 2.** Lifestyle characteristics of study participants (*n* 426) (Numbers and percentages; mean values and standard deviations)

	*n*	%
Live-in household helper		
No	168	39·4
Yes	258	60·6
Presence of electronics in the child’s bedroom		
None	309	72·5
1 or more	117	27·5
Eating while watching TV		
No	149	35
Yes	277	65
Consuming dinner with family		
Never/rarely (includes answer option: Never and 1 or 2 nights)	51	12·1
Sometimes (includes answer option: 3 or 4 nights/5 or 6 nights)	31	7·3
Every night	342	80·7
Use of micronutrient supplements		
No	324	76·1
Yes	102	23·9
Fast-food consumption		
Never/Rarely (includes answer option: Never eats fast food/less than once per months/1–3 times per month)	144	34
Once per week	189	44·7
More than once per week (includes answer option: 2–4 times per week/5–6 times per week/every day)	90	21·2
Breakfast consumption during the past 24 h		
No	10	2·3
Yes	416	97·7
Meeting total physical activity recommendation per week		
No	252	59·2
Yes	174	40·8
Sleep duration (hours)		
Mean	10·37	
sd	7·84	
Healthy Eating Index total score (out of 100)		
Mean	55·15	
sd	18·73	
Poor (score 0–59)	257	60·3
Moderate/good (score 60–100)	169	39·7

Table 3 presents the factor loadings for two identified lifestyle patterns: unhealthy and healthy. Higher fast-food consumption (0·679), eating while watching TV (0·674) and having a live-in household helper (0·445) were positively associated with the unhealthy lifestyle pattern, while having dinner with family (–0·21) showed a negative association. For the healthy lifestyle pattern, meeting physical activity recommendations (0·566), HEI (0·537) and micronutrient supplement use (0·431) had strong positive loadings. The presence of electronics in the child’s bedroom (–0·412) was negatively associated with the healthy lifestyle, while breakfast consumption (0·359) and sleep duration (0·247) had moderate positive associations. (Table 3)

**Table 3.** Factor loadings for the derivation of the lifestyle patterns in the study population (*n* 426)

	Patterns	
	Unhealthy lifestyle	Healthy lifestyle
Fast-food consumption	0·679	–0·142
Eating while watching TV	0·674	0·186
Live-in household helper	0·445	0·107
Consuming dinner with the family	–0·21	0·079
Meeting recommendations for physical activity	0·005	0·566
HEI	–0·126	0·537
Use of micronutrient supplements	0·179	0·431
Presence of electronics in the child’s bedroom	0·33	–0·412
Breakfast consumption	–0·111	0·359
Total sleep hours	0·117	0·247

HEI, Healthy Eating Index.

**Fig. 1.**
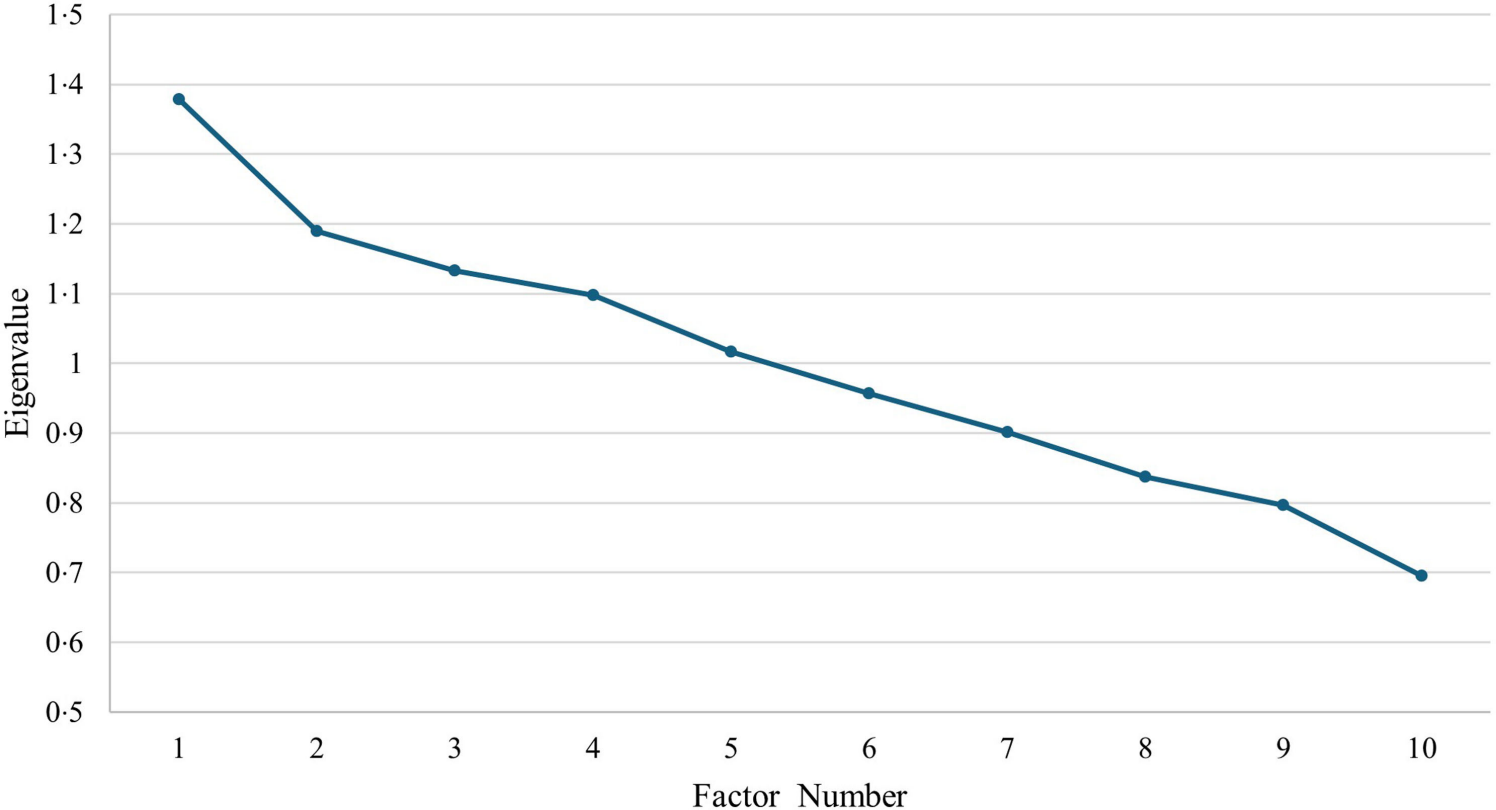
Scree plot.

Figure 1 presents the scree plot, illustrating the eigenvalues associated with each factor/component. The eigenvalues represent the amount of variance explained by each factor. As observed, the first two factors exhibit the highest eigenvalues, signifying that they account for the most significant portion of the total variance in the data. Furthermore, the plot reveals a pronounced inflection point (elbow) between the second and third factors, suggesting that the variance explained by the third and subsequent factors is relatively small as compared with the first and second components (Fig. 1).

Table 4 presents OR for the associations between unhealthy and healthy lifestyle patterns with overweight/obesity in the study population. For the unhealthy lifestyle pattern, both the crude (OR = 1·02; 95 % CI: 0·76, 1·38; *P* = 0·887) and adjusted models (OR = 0·85; 95 % CI: 0·61, 1·20; *P* = 0·356) showed no significant association with overweight/obesity. In contrast, for the healthy lifestyle pattern, the crude model (OR = 0·78; 95 % CI: 0·60, 0·99; *P* = 0·046) and the adjusted model (OR = 0·7; 95 % CI: 0·53, 0·93; *P* = 0·014) indicated a significant negative association, suggesting that a healthy lifestyle reduces the odds of being overweight or obese. (Table 4).

**Table 4.** Odds ratios and their corresponding confidence intervals for the associations of unhealthy (a) and healthy (b) lifestyle patterns with overweight and obesity in the study population (*n* 426)^*^ (Odds ratios and 95 % confidence intervals)

	OR	95 % CI	*P*
Unhealthy lifestyle pattern			
Crude	1·02	0·76, 1·38	0·887
Adjusted^†^	0·85	0·61, 1·20	0·356
Healthy lifestyle pattern			
Crude	0·78	0·60, 0·99	**0·046**
Adjusted^†^	0·7	0·53, 0·93	**0·014**

BAZ, BMI-for-age Z-score.

Bold values indicate significance at *P*-value < 0·05.

* The dependent variable is BAZ categorising participants as overweight or obese *v*. normal (reference category). The normal includes at risk of overweight (for ages 4–5 years) and the wasted categories. The main independent variables are continuous measures representing the healthy and unhealthy lifestyle patterns.

† Basic characteristics associated with overweight/obesity identified in the simple regression analysis were included in the adjusted model. The model was adjusted for the child’s age and sex, the mother’s age and BMI, the mother’s employment status, the father’s education and the crowding index.

## Discussion

To our knowledge, this study is the first to examine the association between lifestyle patterns and paediatric overweight/obesity in the Eastern Mediterranean Region, a region that harbours one of the highest rates of childhood obesity worldwide^(3,58,59)^. Two main lifestyle patterns were identified – the ‘unhealthy’ and the ‘healthy’ patterns, with the latter being associated with significantly lower odds of overweight and obesity in the study sample.

In contrast to traditional methodologies in nutritional epidemiology, the lifestyle pattern approach transcends ‘reductionism in nutrition research’ and offers a holistic lens in the examination of health conditions that are multifactorial in nature, such as overweight and obesity^(60,61)^. Rather than examining each lifestyle factor alone (such as diet, physical activity or sleep, among others) and its association with overweight or obesity, this holistic approach considers the whole lifestyle pattern and the potential circular causalities between the various risk factors^(61)^. Therefore, a lifestyle pattern is not to be viewed as the mere sum of its constitutive factors but is rather perceived as the dynamic interplay between them. This suggests that the impact of a lifestyle pattern would ‘exceed that of any of its parts’ and could therefore capture associations and implications that are closer to real-life conditions^(61)^. In our study, we identified two main lifestyle patterns among children in the UAE, a finding that is in line with several studies conducted on this age group, whereas other studies have identified three patterns: the healthy, the unhealthy and the mixed pattern, the latter including both favourable and unfavourable lifestyle factors^(62)^.

In this study, 11·5 % of children aged 4–9 years in the UAE were overweight or obese, an estimate that is lower than the one reported by a former smaller study performed in the Emirate of Sharjah, where the prevalence rate was estimated at 28·2 % among 6–11-year-old children^(63)^. This same study found that the prevalence of obesity was the highest (14·2 %) in children aged 11 years and the lowest (3 %) at 7 years of age, a finding that is consistent with the results of a larger cross-sectional study among UAE school-aged children^(64)^. Al-Haddad *et al.* reported that the prevalence of overweight and obesity varied across age in the UAE, whereby children below 9 years of age from both sexes were below the Cole *et al.* international overweight and obesity standards, with an increasing prevalence occurring thereafter in 9–18-year-old children^(64)^. This age-dependent gradient may explain the lower prevalence of overweight and obesity observed in our study. The healthy lifestyle pattern identified in our study, which was characterised by higher diet quality (measured by the HEI), physical activity, the use of micronutrient supplements, breakfast consumption, increased hours of sleep and not having electronic devices in the child’s bedroom, was associated with significantly lower odds of overweight/obesity in the study sample, and this protective effect remained significant after adjusting for multiple confounders. This healthy lifestyle pattern confirms the previously reported clustering of favourable health behaviours, such as low screen time, appropriate sleep duration and healthy dietary pattern among children^(65)^, and is in many instances similar to the ‘healthy pattern’ described by D’Souza *et al.* (2020)^(62)^ in their systematic review of lifestyle patterns among children aged 5–12 years. The inclusion of sleep was described by D’Souza *et al.* (2020) as a novel aspect in lifestyle pattern investigations. In agreement with our study approach, two previous studies^(66,67)^ have also included sleep in their lifestyle pattern investigations and reported healthy patterns where high sleep co-existed with a healthy diet, high physical activity and low sedentary behaviour. Our healthy lifestyle pattern also shares many similarities with the ‘low screen time, high sleep and healthy diet’ lifestyle pattern reported by the GECKO Drenthe cohort in the Netherlands^(65)^. Similarly to our findings, data from the GECKO Drenthe cohort showed that children with higher adherence to the ‘low screen time, high sleep and healthy diet’ pattern had significantly lower odds of developing overweight and a lower BMI z-score at 10–11 years^(65)^. Our findings were also consistent with those reported by Aragón-Martín *et al.*, where the adoption of healthier lifestyles and maintaining good physical fitness were shown to be associated with healthier body weight in schoolchildren^(68)^.

The protective influence of the healthy lifestyle pattern identified in our study can be attributed to several factors. Engaging in regular physical activity and consuming a higher quality diet and nutrient-dense foods, such as fruits, vegetables, whole grains and dairy products, may contribute to a healthier energy balance, thereby reducing the risk of overweight and obesity^(69–71)^. In support of our findings, a recent review and meta-analysis of diet quality and obesity among children and adolescents confirmed that a higher adherence to a better quality diet is associated with lower BMI. Further supporting this, a cross-sectional study conducted among schoolchildren (aged 9–13 years) in Greece^(72)^, showed that children belonging to the fourth quartile of a lifestyle pattern characterised by the consumption of high-fibre foods (such as fruits, vegetables and wholegrain products) were significantly less likely to have high adiposity (as assessed the sum of skinfold thicknesses) than those in the first quartile of this pattern^(72)^. Nutrient-dense foods such as fruits, vegetables, whole grains and dairy products are typically rich in dietary fibre, water and essential micronutrients, which promote satiety and help regulate appetite^(73)^. These foods also tend to have lower energy density, enabling individuals to consume adequate food volume with fewer calories, thereby reducing overall energy intake. Furthermore, diets high in fibre and complex carbohydrates can improve glycemic control and reduce insulin spikes, which are associated with fat storage^(74)^.

In line with our findings pertinent to breakfast consumption, the study conducted in Greece^(72)^ showed that children in the fourth quartile of the lifestyle pattern characterised by adequate breakfast consumption and higher dairy foods’ intake were significantly less likely to be overweight/obese than those in the first quartile of this pattern. A recent systematic review and meta-analysis showed that skipping breakfast is associated with a higher risk of overweight/obesity among children, with consistent findings from both cohort and cross-sectional studies^(75)^. Although the mechanisms are still inconclusive, it has been suggested that breakfast skipping may be associated with lower satiety during the day and a higher consumption of high-fat snacks, which can therefore affect appetite regulation, diet quality, energy intake and insulin sensitivity^(76)^. In our study, we included sleep as an additional lifestyle behaviour since shorter sleep duration has also been implicated in the aetiology of childhood obesity^(77)^. Short sleep duration was found to be associated with reduced levels of leptin, insulin sensitivity and glucose tolerance, while being linked to higher levels of ghrelin, appetite and hunger, all of which can promote excessive weight gain in children^(78)^. In line with our findings, a previous study conducted among 6–7-year-old children in Australia^(67)^ showed that those adhering to a ‘short sleep and unhealthy diet’ lifestyle pattern had a significantly higher risk of obesity compared with those adhering to the ‘healthy’ pattern. The absence of electronic devices in the bedroom, another lifestyle factor considered in our study, may translate into lower screen time and lower sedentarity among children, an observation that has been reported by previous studies^(79)^. In line with our results, the ‘low screen time, high sleep and healthy diet’ lifestyle pattern identified among Dutch children was also found to be linked with lower obesity risk^(65)^, whereas another study reported that a ‘high television time, unhealthy eating routines and increased sedentary time’ pattern was linked to childhood overweight^(80)^. Our findings therefore provide additional evidence that screen time and unhealthy diet tend to cluster in a pattern that increases childhood obesity. An interesting finding in our study is the fact that the use of micronutrient supplements was part of the healthy lifestyle pattern. Previous studies have shown that children whose parents encouraged healthier, holistic lifestyles were also more likely to be taking micronutrient supplements^(81)^. Health-conscious parents may be encouraging supplement use if they perceive these supplements as healthy and if they believe that their children cannot meet their micronutrient needs through diet alone^(81)^. It may also be argued that certain micronutrient supplements may play a direct role in preventing excessive adiposity. For instance, calcium and/or vitamin D supplementation were associated with lower visceral adiposity, with potential explanations being linked to the effect of calcium on increasing fat oxidation and thermogenesis via the upregulation of uncoupling proteins and the potential endocrine role of vitamin D^(82)^. The results of this study should not be interpreted as encouraging the use of supplements among children, as it has been clearly established that the diet is the optimal way for children to meet all nutritional requirements^(83)^. The findings of this study rather serve to further confirm that certain lifestyle or behavioural factors tend to cluster together^(81)^. All these factors together, which were part of the healthy lifestyle pattern in our study, were associated with a 30 % reduction in the odds of overweight/obesity in the study sample, thus potentially reflecting synergistic and additive interactions between the various behaviours among children.

In our study, the unhealthy lifestyle pattern was characterised by fast-food consumption, eating meals while watching TV, the presence of a live-in household helper and rarely having dinner with family. It is not surprising that these lifestyle factors are associated with the unhealthy pattern. For instance, frequent consumption of energy-dense foods – such as fast food – was previously shown to be linked with poor dietary quality and low Healthy Eating Index score in children^(84,85)^. Additionally, research showed that eating while watching television promotes mindless eating, higher calorie intake and a lower-quality diet, often characterised by increased consumption of energy-dense, nutrient-poor foods and reduced intake of fruits and vegetables^(86,87)^. The presence of a live-in household helper is a common culture-specific feature of countries in the Eastern Mediterranean Region, and it has been previously found to influence children’s diets as helpers may frequently offer snacks or less healthy food options. This may be due to their limited nutritional knowledge, lack of training in child feeding practices or efforts to satisfy children’s preferences^(88,89)^. In contrast, the role of parents in encouraging healthier dietary practices has been repeatedly described in the literature^(90,91)^, and this may explain why the unhealthy pattern in our study was negatively associated with having family dinners. In our study, the unhealthy pattern was not significantly associated with overweight and obesity among children. This is in contrast to findings reported by a recent systematic review, where unhealthy lifestyle patterns were found to be more often associated with obesity risk than the healthy or mixed patterns. It is essential to recognise that, due to the data-driven nature of factor analysis, the specific lifestyle factors contributing to each pattern may vary across studies^(92)^. Moreover, differences in study findings may result from variations in age groups, the number and labelling of retained patterns and the types of confounders the studies have adjusted for^(93,94)^. In addition, we should not exclude the possibility that measurement limitations or limited variations in exposure may have resulted in the non-significant association between the unhealthy pattern and child overweight in our study.

The study findings ought to be interpreted based on the following limitations. First, the three Emirates chosen for this study collectively represent 85 % of the UAE’s population. Consequently, excluding the remaining 15 % residing in the other four Emirates could be considered a limitation. These Emirates may differ in demographic characteristics, cultural practices and urbanisation levels, all of which can influence key lifestyle factors examined in this study – such as dietary habits, physical activity levels, household structure (e.g. presence of live-in helpers) and screen time behaviours. As a result, the identified lifestyle patterns and their associations with overweight/obesity may not fully reflect those in more rural or less densely populated Emirates. Therefore, caution is warranted when generalising these findings to the national level, and future research should aim to include a more geographically diverse sample across all seven Emirates. In addition, children of mothers under 18 years were excluded from this study, as individuals below this age are considered minors and their recruitment would thus require parental consent, posing logistical challenges. While data on child marriage prevalence in the UAE are unavailable, estimates from culturally similar Gulf Cooperation Council countries – Saudi Arabia, Qatar and Oman – range from 2 to 4 %^(95–97)^, suggesting that this exclusion is unlikely to have significantly impacted sample representativeness. Furthermore, the cross-sectional design of the study primarily allows for examining associations and does not permit to draw causal relationships. More specifically, the observed associations between lifestyle patterns and overweight/obesity may be subject to reverse causation. For instance, it is possible that children with overweight or obesity are more likely to adopt certain lifestyle behaviours (e.g. less physical activity or altered eating habits), rather than these behaviours causing excess weight. Hence, there is a need for future longitudinal studies to confirm the directionality of the study findings. Furthermore, the factor analysis methodology, which is a data-driven approach that tends to identify population-specific patterns^(92)^. Therefore, our findings may reflect patterns that are, in some respects, specific to the children in the UAE. Additionally, factor analysis involves several arbitrary assumptions, including those concerning the number of identified factors and their labels^(93,94,98)^. However, to minimise subjectivity in this approach, the retained factors were selected after evaluating the scree plots and eigenvalues. A related limitation concerns the marginal adequacy of the dataset for factor analysis, as indicated by the Kaiser–Meyer–Olkin value of 0·505. Although the analysis produced interpretable lifestyle patterns, the relatively low intercorrelations among variables suggest that the identified factors should be interpreted with caution. Future research with more strongly correlated lifestyle indicators may help to confirm and refine these patterns. Another limitation arises from using the 24-HR for dietary assessment, a method that may be prone to reporting bias by caregivers^(99)^. Despite its dependence on memory and possible day-to-day variations in consumption patterns, the 24-HR method has been shown to accurately estimate dietary energy intakes at the population level^(100)^. Furthermore, the use of the multiple-pass approach in administering the recalls may mitigate some limitations of the 24-HR^(101)^. To minimise interviewer errors, research nutritionists who conducted the 24-HR interviews underwent extensive training before the initiation of data collection. These nutritionists were also trained to avoid both verbal and non-verbal judgmental cues to reduce social desirability bias. For the 24-HR, using the mother as a proxy to report the child’s food intake may be an additional limitation, as children in this age group (7–9 years) tend to have greater autonomy and may consume food outside the home, beyond the caregiver’s observation. In this study, although several lifestyle and sociodemographic variables were considered, we cannot rule out residual confounding from unmeasured factors such as genetic predisposition, additional behavioural patterns or environmental influences, which may have affected the observed associations. In particular, paternal BMI – an established risk factor for childhood obesity through both genetic predisposition and shared family lifestyle behaviours – was not assessed and could have influenced the observed associations^(102,103)^. In this study, certain lifestyle factors, including parent-reported sleep duration and the number of electronic devices in the child’s bedroom (used as a proxy for screen time), may be subject to recall bias or social desirability bias or may not fully capture actual behaviours. Such measures are commonly applied in studies of this nature and offer reasonable estimates when objective measurements are not available^(104–106)^.

### Conclusion

In conclusion, this study identified two main lifestyle patterns among children aged 4–9 years in the UAE: the healthy and the unhealthy patterns. The results showed that a healthy lifestyle pattern, characterised by better dietary quality, physical activity, increased hours of sleep, breakfast consumption, the use of micronutrient supplements and not having electronic devices in the child’s bedroom, was associated with 30 % lower odds of overweight/obesity in the study sample. These findings highlight the combined impact of various lifestyle behaviours on conditions that have complex, multifactorial aetiologies, such as obesity. The study findings also provide context-specific evidence for health authorities in the UAE to prioritise ‘holistic’ lifestyle modifications when designing culturally sensitive strategies and interventions aimed at preventing obesity among school-aged children. Such evidence-based interventions are crucial for public health, especially considering the harmful impact of obesity on both short- and long-term health outcomes. Public health policies could promote the incorporation of lifestyle interventions into the education and healthcare systems, emphasising approaches grounded in the community’s culture and values. These strategies may include school-based programmes that integrate nutrition and sleep hygiene education into the curriculum, promote physical activity through structured and unstructured play and discourage screen-based sedentary behaviours during and after school hours. Family-centred interventions should also be considered, such as parental workshops and culturally appropriate promotion campaigns that focus on holistic healthy lifestyles targeting caregivers, to improve awareness of healthy diets, sleep and activity practices at home. Collaboration with local stakeholders, including policymakers, educators and media influencers, may enhance reach and community buy-in. Further studies are needed to confirm these findings in other populations. Similarly, future longitudinal studies are warranted to assess the temporal relationships and stability of these lifestyle patterns over time, thereby strengthening the causal interpretation of these findings.

## Supporting information

Naja et al. supplementary material Naja et al. supplementary material

## Supplementary material

For supplementary material/s referred to in this article, please visit https://doi.org/10.1017/S0007114525105242
