# Supplementary material for: Lifestyle patterns and their associations with overweight and obesity among children aged 4–9 years in the United Arab Emirates
Source: Br J Nutr. 2025 Sep 26;134(9):733–42. doi: 10.1017/S0007114525105242 (PMC12766110; doi:10.1017/S0007114525105242)
Supplement: Naja et al. supplementary material [file S0007114525105242sup001.docx]

**Table A.1.** Healthy Eating Index (HEI)-2020 components and scoring standards for children ages 2 and older

| **Component** | **Maximum**  **points^a^** | **Standard for maximum score** | **Standard for minimum score of zero** |
| --- | --- | --- | --- |
| **Adequacy Components** |  |  |  |
| Total Fruits^b^ | 5 | ≥ 0.8 cup equiv. per 1,000 kcal | No Fruit |
| Whole Fruits^c^ | 5 | ≥ 0.4 cup equiv. per 1,000 kcal | No Whole Fruit |
| Total Vegetables^d^ | 5 | ≥ 1.1 cup equiv. per 1,000 kcal | No Vegetables |
| Greens and Beans | 5 | ≥ 0.2 cup equiv. per 1,000 kcal | No Dark Green Vegetables or Legumes |
| Whole Grains | 10 | ≥ 1.5 oz equiv. per 1,000 kcal | No Whole Grains |
| Dairy^e^ | 10 | ≥ 1.3 cup equiv. per 1,000 kcal | No Dairy |
| Total Protein Foods^d^ | 5 | ≥ 2.5 oz equiv. per 1,000 kcal | No Protein Foods |
| Seafood and Plant Proteins^f^ | 5 | ≥ 0.8 oz equiv. per 1,000 kcal | No Seafood or Plant Proteins |
| Fatty Acids^g^ | 10 | (PUFAs + MUFAs) / SFAs ≥ 2.5 | (PUFAs + MUFAs) / SFAs ≤ 1.2 |
| **Moderation Components** |  |  |  |
| Refined Grains | 10 | ≤ 1.8 oz equiv. per 1,000 kcal | ≥ 4.3 oz equiv. per 1,000 kcal |
| Sodium | 10 | ≤ 1.1 grams per 1,000 kcal | ≥ 2.0 grams per 1,000 kcal |
| Added Sugars | 10 | < 6.5% of energy | ≥ 26% of energy |
| Saturated Fats | 10 | ≤ 8% of energy | ≥ 16% of energy |

Abbreviations: MUFAs, monounsaturated fatty acids; PUFAs, polyunsaturated fatty acids; SFAs, saturated fatty acids.

^a^ Between the minimum and maximum standards are scored proportionately.

^b^ Includes 100% fruit juice.

^c^ Includes all forms except juice.

^d^ Includes beans, peas, and lentils.

^e^ Includes all milk products, such as fluid milk, yogurt, and cheese, and fortified soy beverages.

^f^ Includes seafood, nuts, seeds, soy products (other than beverages), and beans, peas, and lentils.

^g^ Ratio of PUFAs and MUFAs to SFAs.
